# Chronic restraint stress induces hippocampal memory deficits by impairing insulin signaling

**DOI:** 10.1186/s13041-018-0381-8

**Published:** 2018-07-03

**Authors:** Hanwoong Woo, Caroline Jeeyeon Hong, Seonghee Jung, Seongwon Choe, Seong-Woon Yu

**Affiliations:** 1 Department of Brain and Cognitive Sciences, Daegu Gyeongbuk Institute of Science and Technology (DGIST), 333 Techno Jungang Daero, Hyeonpung-Myeon, Dalseong-Gun, Daegu, 42988 Republic of Korea; 2 Neurometabolomics Research Center, Daegu Gyeongbuk Institute of Science and Technology (DGIST), Daegu, 42988 Republic of Korea

**Keywords:** Chronic restraint stress, Corticosterone, Insulin signaling, Intranasal insulin delivery, Nesting, Y-maze

## Abstract

Chronic stress is a psychologically significant factor that impairs learning and memory in the hippocampus. Insulin signaling is important for the development and cognitive function of the hippocampus. However, the relation between chronic stress and insulin signaling at the molecular level is poorly understood. Here, we show that chronic stress impairs insulin signaling in vitro and in vivo, and thereby induces deficits in hippocampal spatial working memory and neurobehavior. Corticosterone treatment of mouse hippocampal neurons in vitro caused neurotoxicity with an increase in the markers of autophagy but not apoptosis. Corticosterone treatment impaired insulin signaling from early time points. As an in vivo model of stress, mice were subjected to chronic restraint stress. The chronic restraint stress group showed downregulated insulin signaling and suffered deficits in spatial working memory and nesting behavior. Intranasal insulin delivery restored insulin signaling and rescued hippocampal deficits. Our data suggest that psychological stress impairs insulin signaling and results in hippocampal deficits, and these effects can be prevented by intranasal insulin delivery.

## Introduction

Stress affects various parts of our body and causes diverse physiological changes, which are manifested in symptoms such as headache, stomachache, heartburn, fatigue, overeating, or undereating. Severe and long-lasting adverse effects induced by stress include insomnia, anxiety, depression, or post-traumatic stress disorder [1, 2]. These physiological outcomes of psychological stress are mainly caused by stress hormones [2]. Stress stimulates the hypothalamus to release corticotropin-releasing hormone (CRH) into the portal vein. CRH induces anterior pituitary to release adrenocorticotrophic hormone (ACTH). ACTH affects the adrenal cortex and increases synthesis and release of corticosteroids. Corticosteroids include glucocorticoids, which regulate glucose metabolism, and mineralocorticoids, which regulate water balance and blood pressure. The major glucocorticoid is cortisol in humans and corticosterone (CORT) in rodents. CORT delivered with blood causes diverse stress responses in tissues and returns to the hypothalamus and pituitary for a negative feedback on CRH and ACTH secretion [3–6].

To recapitulate psychological stress in animal models, several procedures have been developed, including unpredictable chronic stress, chronic restraint stress (CRS), and chronic administration of CORT [7–9]. CRS has been widely used as a model of chronic psychoemotional stress to induce depressive- and anxiety-like behaviors, learning and memory deficits, and hippocampal neuronal damage in mice [10, 11]. One of the most vulnerable targets of stress is hippocampus, because it abundantly expresses both glucocorticoid and mineralocorticoid receptors [12, 13]. Stress changes hippocampal neural activity and synaptic plasticity, activates hippocampal glucocorticoid receptor (GR), and decreases neuronal cell survival and neurogenesis [14–16]. Prolonged exposure to CORT also causes hippocampal neuronal damage and impairs hippocampal neurogenesis, synaptic plasticity, and learning in neuronal cultures and in mice [17–19].

Insulin signaling is important for the development and cognitive function of the hippocampus [20–22]. Insulin receptor (IR) is abundantly distributed in the hippocampus, and insulin binds to IR to initiate insulin/IR signaling [23]. Insulin signaling mediates neuronal development, feeding behavior, and cognitive processes [24]. Attenuated insulin production and IR activity result in learning and memory formation deficits [25], and deletion of brain IR leads to augmented anxiety, depressive-like behavior, and deficits in long-term memory [26, 27]. Blockade of IR or downstream signaling molecules such as phosphatidylinositol-3-kinase (PI3K) impairs hippocampal memory function [28, 29], whereas intrahippocampal insulin microinjection improves spatial memory [29].

Activated IR recruits and phosphorylates substrate adaptors such as the family of insulin receptor substrate (IRS) proteins. IRS-1 is a major IR substrate and a key mediator in insulin signaling. IRS-1 acts as a docking protein between the IR and intracellular signaling molecules that mediate metabolism and growth [30]. PI3K binds to activated IRS proteins and activates Akt kinase, which plays a critical role in cell survival. Akt also activates mammalian target of rapamycin (mTOR), which promotes protein synthesis and attenuates autophagy [31–33]. To target the insulin signaling pathway for the improvement of cognitive function, previous studies have tested intranasal insulin delivery to healthy subjects and showed an improvement in learning and memory in mice [34, 35] and in humans [36, 37]. However, only a few studies associated CORT with insulin resistance in the brain [38–40], and our understanding of the mechanisms of the effects of CORT on hippocampus, especially on insulin signaling in the hippocampus, is still lacking. Also, so far, there have been only a limited number of studies that examined the protective effects of insulin signaling potentiation against stress-induced hippocampal impairment [41].

Another unanswered question is how CORT induces neurotoxicity. One possible mechanism is that it induces apoptosis [17, 42]. However, it has not been thoroughly examined whether other modes of cell death, such as autophagic cell death, are also involved in CORT-induced neurotoxicity. Autophagy (self-eating) is an essential cellular process characterized by bulk degradation of unnecessary or dysfunctional intracellular components at basal state and under physiologically stress [43]. Double-membrane structures, autophagosomes, engulf portions of the cytosol containing intracellular components and fuse with lysosomes to form autolysosomes where cargoes are degraded by acidic lysosomal hydrolases [44]. Autophagy helps cells to cope with stress by providing metabolic intermediates and thereby contributes to cell survival [44, 45]. On the other hand, excessive autophagy can lead to cell death [46, 47]. Autophagic cell death is defined as cell death with increased autophagic flux without the features of apoptosis or necrosis, and when cell death is prevented by suppression of autophagy [48–50].

Type II microtubule-associated protein light chain 3 (LC3-II) is a well-known biochemical marker of autophagy [51, 52]. LC3 is proteolytically cleaved immediately after its synthesis and becomes LC3-I. Cytosolic LC3-I is recruited to the autophagosome membrane, where it is conjugated with phosphatidylethanolamine and is converted into LC3-II [53]. The ubiquitin-binding protein p62 binds LC3-II and serves as a linker between LC3 and cargoes. Therefore, p62 is degraded together with the cargo molecules and a decrease in p62 level can be another marker of autophagy flux [54].

In this study, we show that CRS impairs insulin signaling in the mouse hippocampus and thereby induces deficits in hippocampal function, whereas intranasal insulin delivery prevents insulin signaling impairment and repairs hippocampal deficits. CORT treatment of primary hippocampal neurons in vitro recapitulates the impairment of insulin signaling with an increase in autophagy.

## Methods

### Animals and CRS procedure

All procedures that involved laboratory animals were approved by the Institutional Animal Care and Use Committee at the DGIST.

Eight-week-old male C57BL/6 N mice were housed with a 12:12 h light-dark cycle (lights on 07:00 to 19:00) with 22–24°C temperature, 40–60% humidity, and food and water were supplied ad libitum. Four male mice were housed in each cage until six-weeks old, and individually housed for a week and handled daily for acclimation for another week before experiments. Mice were randomly divided into the control (Ctrl) and stressed groups. Mice of the stressed group were horizontally immobilized for 6 h/day (from 10:00 to 16:00) in the acrylic cylindrical flat-bottom head-first restrainer (Φ25 × {H}90 mm, Jeungdo Bio & Plant Co., Seoul, Korea) for 2 weeks in their home cages. Restrainer has several slots to restrain the mouse firmly according to the size of each mouse, and suppresses the physical movement of the limbs without causing pain. After being restrained, mice were released back into their home cages immediately. Non-restraint mice (Ctrl) remained in their home cages without CRS procedure, and both Ctrl and CRS mice could not access food and water during the period of CRS exposure. The weight of the mice was measured every week before the experiment.

### Reagents and antibodies

Corticosterone (Sigma-Aldrich, 27840), staurosporine (STS; Cell Signaling Technology, 9953), bafilomycin A1 (Sigma-Aldrich, B1793), insulin-FITC (Sigma-Aldrich, I3661), methyl cellulose (Sigma-Aldrich, M0512), sodium borohydride (Sigma-Aldrich, 213462), and insulin (Roche Diagnostics, 11 376 497 001) were purchased from the indicated companies. Antibodies against IR subunit β (3025), p-Akt-S473 (9271), Akt (9272), p-mTOR-S2448 (2971), mTOR (2972), and cleaved caspase-3 (C.Casp-3; 9664) were purchased form Cell Signaling Technology. Antibodies against p-IRS1-Y612 (Invitrogen, 44-816G), IRS1 (Invitrogen, PA1–1057), GR (Santa Cruz Biotechnology, SC-56851), LC3 (Novus, NB100–2220), p62 (Sigma-Aldrich, P0067) and β-actin-HRP (Santa Cruz Biotechnology, SC-47778) were purchased from the indicated companies.

### Hippocampal neuron culture

Primary hippocampal neurons were obtained from C57BL/6 N mice on embryonic day 17. Briefly, hippocampi were dissociated in Hank’s Balanced Salt Solution (Invitrogen, 14175–095) and maintained in a Neurobasal Medium (Gibco, 21103–049) supplemented with 100 U/ml penicillin, 100 μg/ml streptomycin (HyClone, SV30010), 2 mM L-glutamine (Invitrogen, 25030–081), and 2% B27 supplement (Invitrogen, 17504–044). After 3 days, one-third of the medium was replaced with medium containing cytosine β-D-arabinofuranoside (Sigma-Aldrich, C1768; final concentration, 3 μM). After 4 days, one-third of the medium was replaced with fresh medium without cytosine β-D-arabinofuranoside and cultures were used for experiments on day 11 or 12 days.

### Cell death assay

Cell death was assessed by dual staining of cells with the membrane-permeable dye Hoechst 33342 (Invitrogen, H1399) to stain all cells and with the membrane-impermeable dye propidium iodide (PI; Sigma-Aldrich, P4170) to stain dead cells. The percentage of cell death (%) was calculated by dividing the number of red-positive (PI) cells by that of blue-positive (Hoechst 33342) cells.

### Confirmation of intranasal delivery of insulin into the hippocampus using insulin-FITC

Insulin-FITC reconstituted in 0.01 N HCl was diluted in 0.9% saline with 0.001% methyl cellulose and was delivered twice into both nares: the first dose 6 h and the second dose at 1 h before sacrifice (2.5 μg/24 μl each). Control animals received 0.9% saline with 0.001% methyl cellulose.

### Intranasal delivery of insulin

Mice were hand-restrained in a supine position with the neck in extension and were not anesthetized. A total of 100 μg of insulin in 20 μl of vehicle (0.9% saline with 0.001% methyl cellulose) or the same volume of vehicle alone was delivered daily into both nares simultaneously 30 min before restraint stress for 7 days.

### Nest-building assay

Two nestlets were added in each cage at 16:00 on the 13th day after restraint stress and scores were measured on the 14th day (09:50) before the start of restraint stress.

### Y-maze assay

Y-maze test was used to evaluate spatial working memory of the mice. The test was performed in a Y-shaped maze with three white opaque plastic arms. Mice were placed into the center of the maze and were allowed to explore the three arms freely for 6 min. Each experiment was video recorded with EthoVision software (Noldus, EthoVision XT 11.5). After exploration, alternation (%) was calculated as the number of three consecutive arm entries divided by the number of possible alternations (total arm entries minus two).

### CORT level measurement

Mouse blood serum samples were acquired on the 14th day 30 min after the end of stress from the submandibular vein using animal lancets. Blood CORT levels were measured using an ELISA kit (Enzo Life Sciences, ADI-900-097).

### Preparation of hippocampal lysates

Mice were deeply anesthetized by injecting Zoletil (40 mg/kg) and Rompun (5 mg/kg) and sacrificed by decapitation. Brains were removed, dissected, and hippocampi were isolated on ice and stored in liquid nitrogen. Hippocampi were lysed in radioimmunoprecipitation assay (RIPA) buffer (Sigma-Aldrich, R0278) containing 1 mM phenylmethanesulfonyl fluoride (PMSF; Sigma-Aldrich, 78830), 1 mM dithiothreitol (DTT; Sigma-Aldrich, D9779), 1× protease (Thermo Scientific, 87786) and phosphatase (Thermo Scientific, 78420) inhibitor cocktails by using a 1 ml syringe with 10 strokes, and were further disrupted with Bioruptor (Cosmobio, KRB-01) for 20 min. Following centrifugation (12,000 ×*g*, 10 min), the supernatants were harvested and the protein concentrations were measured using a BCA protein assay reagent (Thermo Scientific, 23224).

### Western blotting analysis

Harvested hippocampal neurons were lysed in RIPA buffer containing 1 mM PMSF, 1 mM DTT and 1× protease and phosphatase inhibitor cocktails for 20 min on ice. Following centrifugation (12,000 ×*g*, 10 min), protein concentrations were measured using the BCA protein assay reagent. Typically, 30 μg of total protein of hippocampal tissue or neuron lysates was loaded per well. Proteins were electro-transferred to polyvinylidene fluoride membranes (Millipore, IPVH00010) in a semidry electrophoretic transfer cell (Bio-Rad). The membranes were incubated overnight with primary antibodies. After washing, the membranes were incubated for 1 h at room temperature with peroxidase-conjugated secondary antibodies. After washing, the membranes were processed for analysis using a chemiluminescence detection kit (Thermo Scientific, 34080).

### Immunocytochemistry

Mouse hippocampal neurons were fixed with 4% paraformaldehyde (PFA) and permeabilized with 0.1% Triton X-100 in phosphate buffered saline (PBS) containing 0.5% bovine serum albumin (BSA; Affymetrix/USB, 10857). Following blocking with 0.5% BSA, the samples were incubated overnight with cleaved caspase-3 antibody at 1:1000 dilutions and then with an anti-rabbit Alexa 488 secondary antibody (Jackson ImmunoResearch, 711–545-152). Samples were stained with Hoechst 33342 and visualized under a confocal laser scanning microscope (Carl Zeiss, LSM 700); images were analyzed using ZEN12 software (Carl Zeiss).

### Tissue histology

Mice were anesthetized by Zoletil and Rompun injection and perfused with PBS, followed by 4% PFA. Brains were then removed, post-fixed in 4% PFA for 12 h and cryoprotected in 30% sucrose until they sank to the bottom of the tube. For fluorescence microscopy, brains were frozen in optimal cutting temperature compound and cryosectioned coronally with a 30 μm thickness. Brain section coordinates were based on the mouse brain atlas [55]. The sections were free-floated in PBS, blocked with 0.1% sodium borohydride in PBS to reduce background autofluorescence, counter stained with Hoechst 33342, and mounted. FITC-tagged insulin was visualized by a slide scanner (Zeiss, Axio Scan.Z1) and images were analyzed using ZEN12 software.

### Statistical analysis

All data were obtained from at least three independent experiments and are presented as mean values ± standard error of the mean (SEM). Statistical significance was determined using one-way analysis of variance (ANOVA) and Tukey’s post-test. Unpaired t test, nonparametric Mann-Whitney U test, or two-way ANOVA and Bonferroni post-test were also used in experiments, as indicated in the figure legends. Differences were considered statistically significant at *p* < 0.05.

## Results

### CORT is neurotoxic and induces autophagy features in mouse hippocampal neurons in vitro

**Fig. 1:**
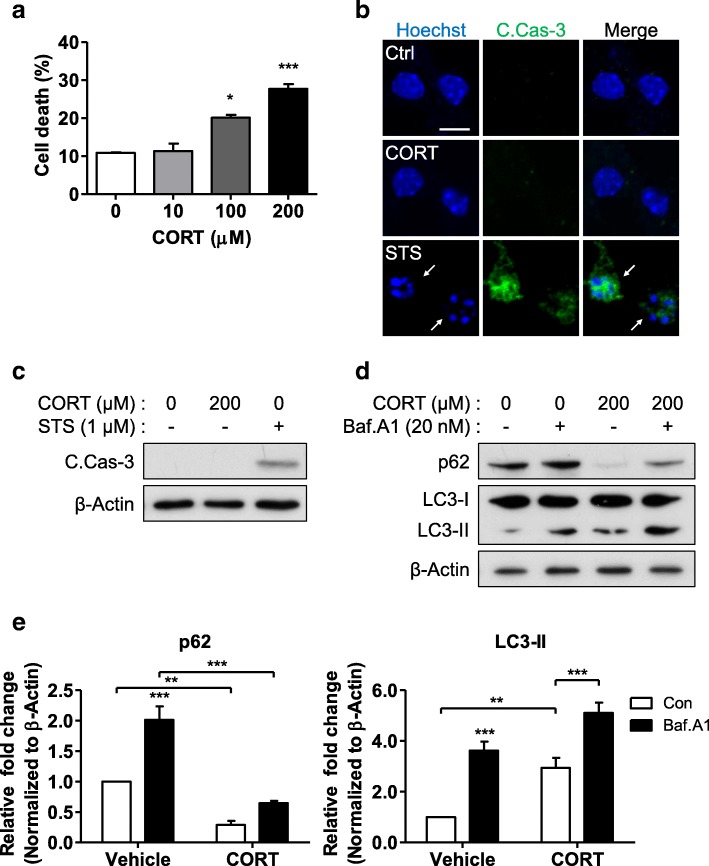
Neurotoxicity of CORT in primary mouse hippocampal neurons. **a** Dose dependence analyzed by one-way ANOVA (*n* = 3). **b** Immunostaining for cleaved caspase-3 (C.Casp-3) after treatment with CORT for 24 h or staurosporine (STS) for 6 h. Arrows indicates apoptotic, condensed nucleus. Scale bar, 10 μm. **c** Western blotting analysis of C.Casp-3 after treatment with CORT for 24 h or STS for 6 h. **d** Western blotting analysis of LC3-II after CORT treatment for 24 h. Bafilomycin A1 (Baf.A1; 20 nM) was added 2 h before sampling. **e** Quantification of p62 and LC3-II analyzed by two-way ANOVA (*n* = 8). Data are mean ± SEM. *p < 0.05, **p < 0.01, ***p < 0.001

Chronic psychological stress or long-term application of CORT results in time-dependent hippocampal neuronal damage [17–19, 38, 42]. However, the underlying mechanisms of stress-induced hippocampal neuronal damage are still not well understood. To examine the neurotoxicity of CORT, we treated mouse hippocampal neurons in vitro with a wide range of CORT doses and found that 100 or 200 μM CORT significantly increased hippocampal neuronal cell death (Fig. 1a). Because there were reports that glucocorticoids induced neuronal apoptosis [17, 42], we examined whether the CORT-induced neurotoxicity was apoptotic or not. We used STS, a widely used apoptosis inducer, as a positive control of apoptosis [56]. C.Casp-3 is active form of caspase-3 and is the best-known marker of apoptosis [57]. Of interest, STS but not 200 μM CORT induced robust C.Casp-3 immunoreactivity (Fig. 1b). Moreover, nuclear condensation, another well-known characteristic of apoptosis, was observed in STS-treated but not in CORT-treated cells (Fig. 1b, arrow). Immunoblot analysis showed that treatment with STS but not with 200 μM CORT induced C.Casp-3 (Fig. 1c). Since apoptotic markers were not detected after CORT treatment, we wondered whether another mode of programmed cell death, such as autophagic cell death [44, 48, 58], is involved. CORT increased the level of LC3-II (Fig. 1d, e). Bafilomycin A1 (Baf.A1) is a well-known inhibitor of the late phase of autophagy and is used to prevent maturation of autophagic vacuoles by inhibiting fusion between autophagosomes and lysosomes [59]. Although an increase in LC3-II level is typically regarded as an indicator of increased autophagy flux, impaired autophagy flux and blockage of autophagic degradation can also result in LC3-II accumulation level, because LC3-II itself is degraded by autophagy. These possibilities can be distinguished by blocking the late phase of autophagy flux. Further accumulation of LC3-II after treatment Baf.A1 would indicates an increase in autophagic flux, not the suppression of autophagic degradation [52, 56]. On the other hand, if the LC3-II level is not significantly affected by Baf.A1, it may suggest impaired autophagy flux. Since p62 is also degraded in autolysosomes, blocking of autophagosome fusion with lysosomes or inhibition of lysosomal hydrolases can cause p62 accumulation under the conditions of high autophagy flux. Baf.A1 treatment of control cells for 2 h led to accumulation of p62 and LC3-II (Fig. 1d, e). CORT treatment increased LC3-II and decreased p62, and the CORT+Baf.A1 group accumulated p62 together with a higher increase in LC3-II than in the CORT-alone group (Fig. 1d, e). These data suggest that CORT does not induce apoptosis, but enhances autophagy flux in mouse hippocampal neurons, suggesting the involvement of autophagic cell death in CORT-induced neurotoxicity.

### CORT impairs insulin signaling in mouse hippocampal neurons in vitro

**Fig. 2:**
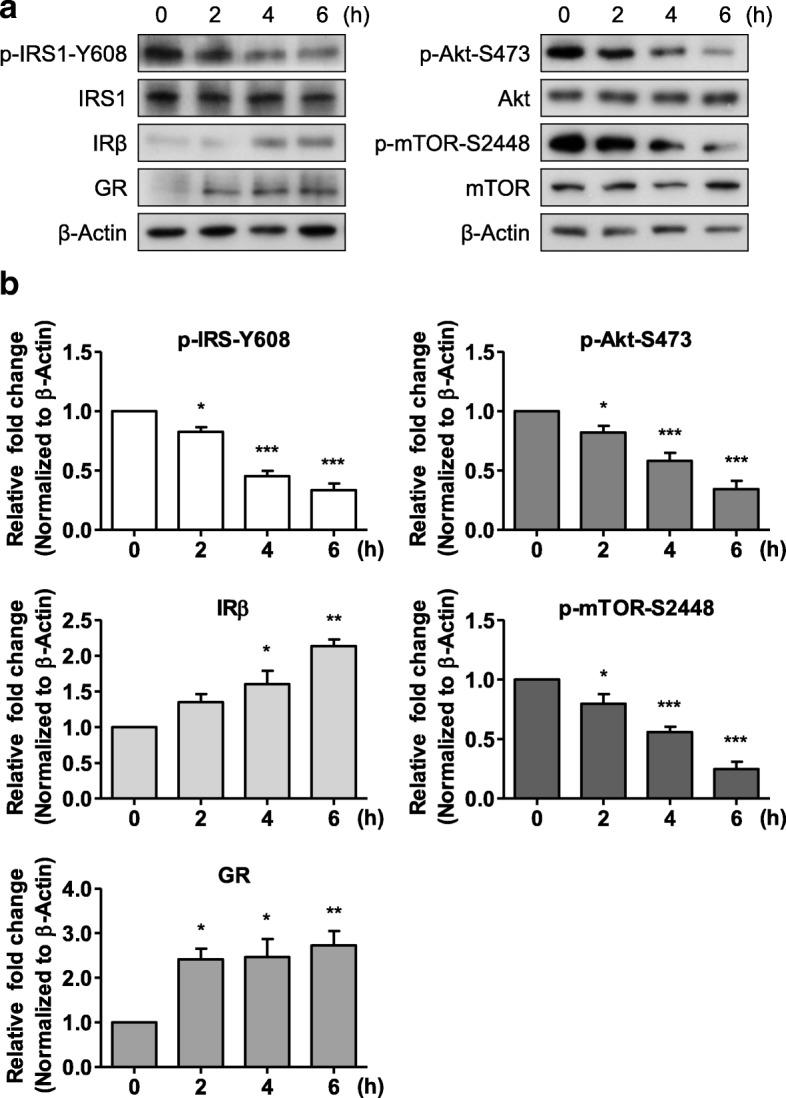
Impairment of the insulin signaling pathway following CORT treatment in primary mouse hippocampal neurons. **a** Western blotting analysis of insulin signaling molecules. **b** Quantification of insulin signaling molecules analyzed by one-way ANOVA (*n* = 4). Blots are representative of four experiments with similar results. Data are mean ± SEM. *p < 0.05, **p < 0.01, ***p < 0.001

Next, we examined whether CORT affects insulin signaling, since insulin signaling is closely related to autophagy and is also important for hippocampal development and function, and for survival of hippocampal neural stem cells and neurons [47, 48, 60]. Treatment of mouse hippocampal neurons with 200 μM CORT increased the expression of GR, a positive marker of CORT action (Fig. 2a, b). CORT treatment downregulated phosphorylation of IRS1 on Y608 (Y612 in humans) without changing the total IRS1 level (Fig. 2a, b). The phosphorylation of Akt on S473 and mTOR on S2448 was also decreased after CORT treatment (Fig. 2a, b). Intriguingly, the expression of IR subunit β (IRβ) was increased by CORT treatment (Fig. 2a, b), which might be a compensation to overcome the impaired insulin signaling [48]. Taken together, these results suggest that CORT impairs insulin signaling (the IRS1–Akt–mTOR pathway) in mouse hippocampal neurons.

### CRS impairs insulin signaling in the mouse hippocampus in vivo

**Fig. 3:**
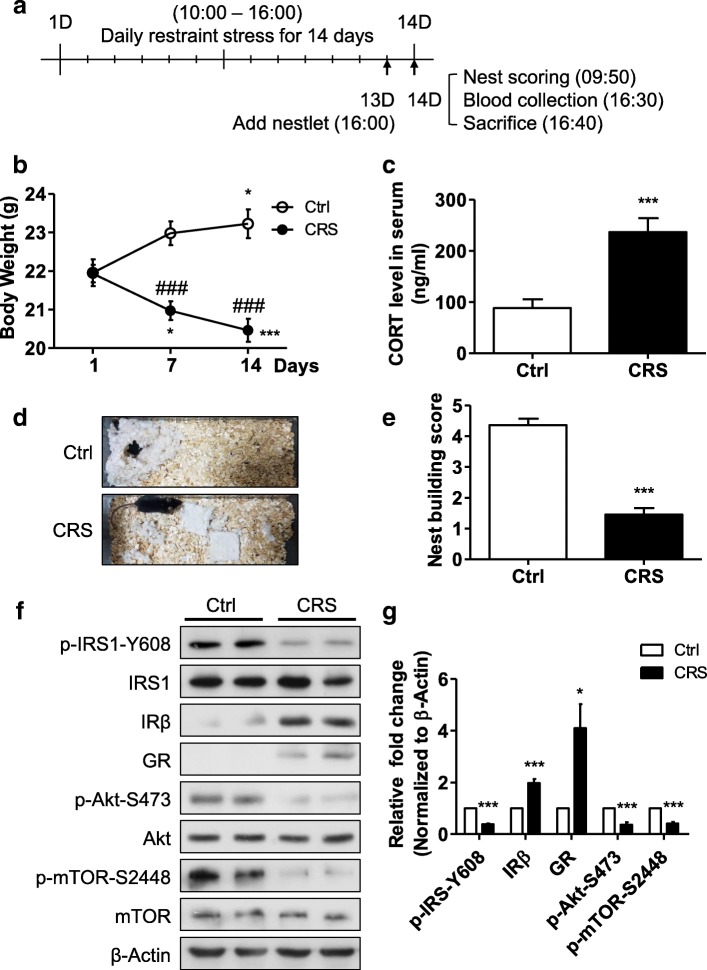
Impairment of insulin signaling pathways and nesting behavior in mice subjected to chronic restraint stress (CRS). **a** Experimental scheme. **b** Body weight analyzed by two-way ANOVA (*n* = 11 per group). *p < 0.05, ***p < 0.001 (difference between days in the same groups); ###*p* < 0.001 (difference between Ctrl and CRS groups on the same days). **c** Concentration of blood CORT analyzed by unpaired t test (*n* = 6). **d** Nest building behavior assay. Representative results are shown. **e** Nest building scores analyzed by nonparametric Mann-Whitney U test (*n* = 11 per group). **f** Western blotting analysis of the insulin signaling pathway in the mouse hippocampus (*n* = 7 per group). **g** Quantification of insulin signaling molecules analyzed by unpaired t test. Data are mean ± SEM. *p < 0.05, ***p < 0.001

Next, we wondered whether psychological stress, such as CRS, which involves high levels of corticoid hormones, affects insulin signaling in vivo. To generate CRS, we used a flat bottom restrainer for 6 h/day for 2 weeks (Fig. 3a). First, we measured stress responses during and after CRS. Whereas the initial body weight did not differ between the CRS and Ctrl groups, CRS significantly inhibited body weight gain compared to Ctrl (Fig. 3b) and significantly increased the circulating blood CORT level after 2 weeks (Fig. 3c). We used nesting behavior as another index of neuronal stress. Nesting behavior depends on normal hippocampal function and is impaired by hippocampal lesions [61]. Nesting behavior was measured by scoring the quality of the nest with a 5-point rating scales. Rating scale 1 means that nestlets are not touched, and 5 means a nearly perfect nest with a crater and higher walls. Unlike the Ctrl group, the CRS group had difficulties in building a nest with nestlets (Fig. 3d, e).

To determine whether CRS influences insulin signaling in the hippocampus, we measured the expression and phosphorylation of relevant signaling proteins by immunoblotting (Fig. 3f, g). The expression of GR was increased in the hippocampi of the CRS group, which is likely a response to an increased blood CORT level. Similar to hippocampal cultures treated with CORT, CRS increased the expression of IRβ. Interestingly, the levels of p-IRS1-Y608, p-Akt-S473 and p-mTOR-S2448 were notably decreased in the CRS group. In summary, these data demonstrate successful induction of stress and subsequent impairment of insulin signaling in the mouse hippocampus following 2 weeks of CRS.

### Intranasal insulin delivery reduces CRS-induced memory impairment in mice

**Fig. 4:**
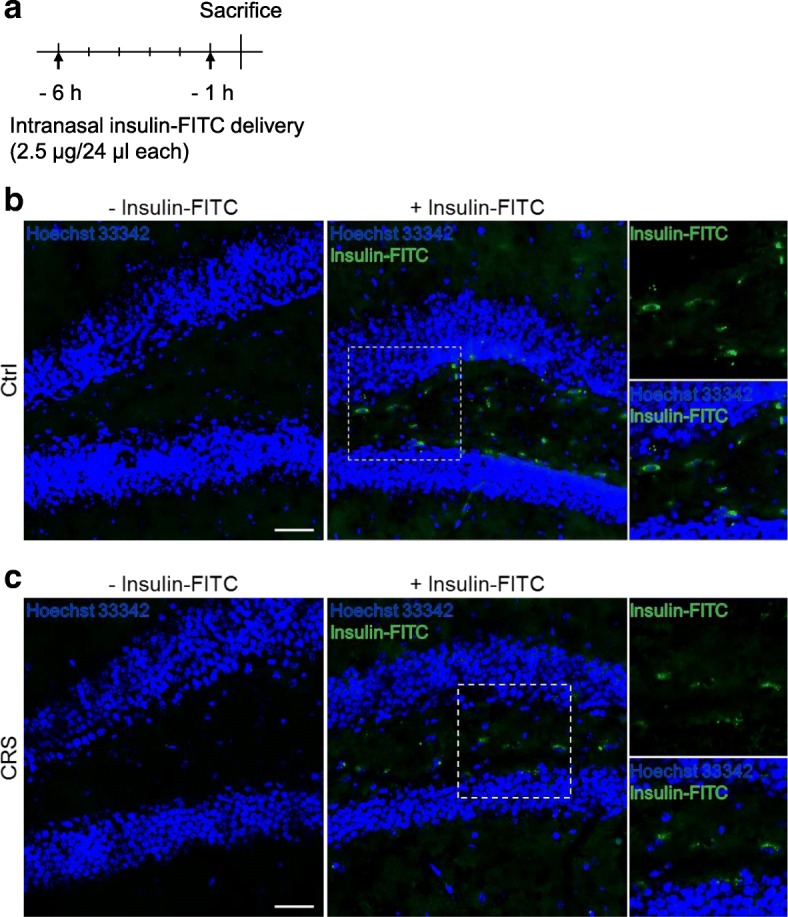
Detection of insulin-FITC after intranasal delivery. **a** Experimental scheme. **b** Representative images of hippocampal tissues without and with insulin-FITC in Ctrl mice (*n* = 3 per group). Anterior-posterior coordinates of images according to mouse brain atlas are Bregma − 1.90 mm (− Insulin-FITC) and − 2.14 mm (+ Insulin-FITC) respectively. Scale bar, 50 μm. **c** Representative images of hippocampal tissues without and with insulin-FITC in CRS mice (*n* = 3 per group). Anterior-posterior coordinates of images according to mouse brain atlas are Bregma − 1.96 mm (− Insulin-FITC) and − 2.26 mm (+ Insulin-FITC) respectively. Scale bar, 50 μm

**Fig. 5:**
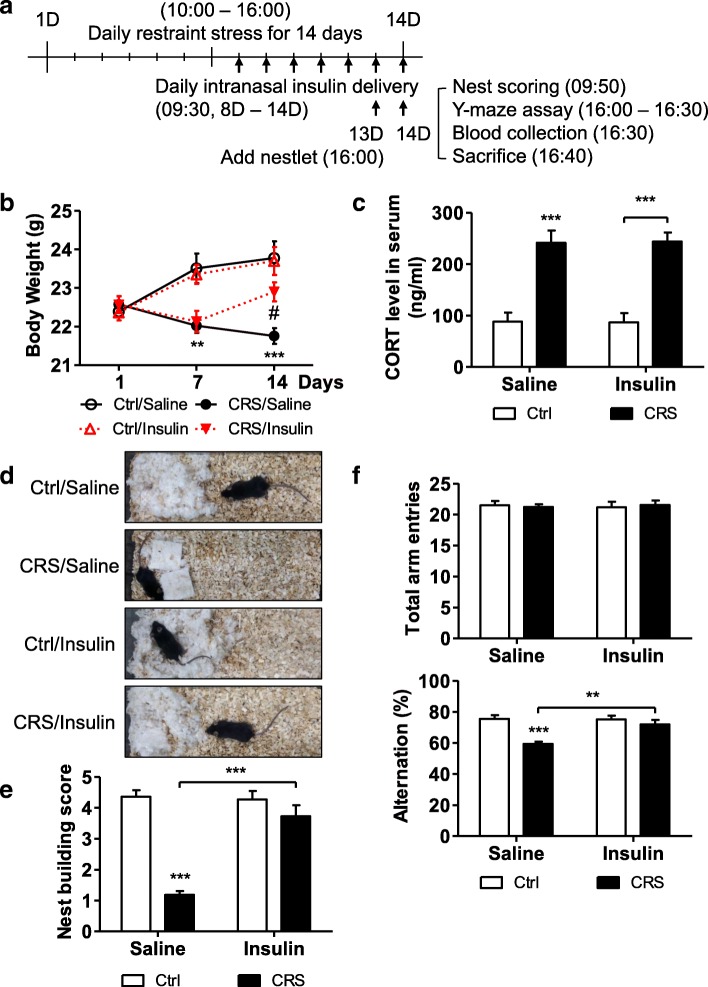
Mitigation of CRS-induced hippocampal damage by intranasal insulin delivery. **a** Experimental scheme. **b** Body weight analyzed by two-way repeated ANOVA (*n* = 9 per group). **p < 0.01, ***p < 0.001 (difference between Ctrl/Saline and CRS/Saline groups on the same days); #*p* < 0.05 (difference between CRS/Saline and CRS/Insulin groups on the same days). **c** Concentration of blood CORT analyzed by two-way ANOVA (Ctrl/Saline, *n* = 6; Ctrl/Insulin, *n* = 6; CRS/Saline, *n* = 7; CRS/Insulin, *n* = 7). **d** Nest building behavior assay. Representative results are shown. **e** Nesting building scores analyzed by two-way ANOVA (bottom) (*n* = 11 per group). **f** Y-maze test analyzed by two-way ANOVA (Ctrl/Saline, *n* = 6; Ctrl/Insulin, *n* = 6; CRS/Saline, *n* = 9; CRS/Insulin, *n* = 9). Data are mean ± SEM. **p < 0.01, ***p < 0.001. Ctrl/saline, control group with saline; CRS/saline, CRS group with saline; Ctrl/insulin, control group with insulin administration; CRS/insulin, CRS group with insulin administration

To test whether down-regulated insulin signaling can be recovered by insulin delivery to the hippocampus, we delivered insulin intranasally. First, to confirm that insulin arrived into the hippocampus, we tagged insulin with FITC and tracked it by histology image analysis after intranasal delivery (Fig. 4a). Administration of insulin-FITC but not saline resulted in green fluorescence in the dentate gyrus areas of the hippocampus 6 h after administration (Fig. 4b). Administration of insulin-FITC in CRS mice showed similar green fluorescence distribution (Fig. 4c). These data validated successful delivery of insulin into the hippocampus in both Ctrl and CRS mice. Next, we divided mice into 4 groups: Ctrl with saline (Ctrl/Saline), CRS with saline (CRS/Saline), Ctrl with insulin (Ctrl/Insulin), and CRS with insulin (CRS/Insulin). Insulin or saline was intranasally delivered for 7 days during the 2nd week of CRS (Fig. 5a). Both Ctrl/Saline and Ctrl/Insulin groups showed an increased in body weight, while the CRS/Saline group showed significantly reduced body weight (Fig. 5b). Interestingly, the CRS/Insulin group also showed reduced body weight after 1-week-CRS, but the reduction was reversed after intranasal administration of insulin (Fig. 5b). This shows a potential beneficial effect of intranasal insulin delivery for the functional and metabolic recovery of stressed hippocampus. Blood CORT level and nest-building behavior were also measured after experiments. Interestingly, intranasal administration of insulin did not alter the level of CORT increased by CRS (Fig. 5c). This data suggest that the neuroprotective activity of insulin is by suppressing autophagy in the hippocampus against the detrimental effects of CORT, but not by alteration of the Hypothalamic-Pituitary-Adrenal axis. The CRS/Insulin group also regained the ability to build a nest compared to the CRS/Saline group (Fig. 5d, e).

**Fig. 6:**
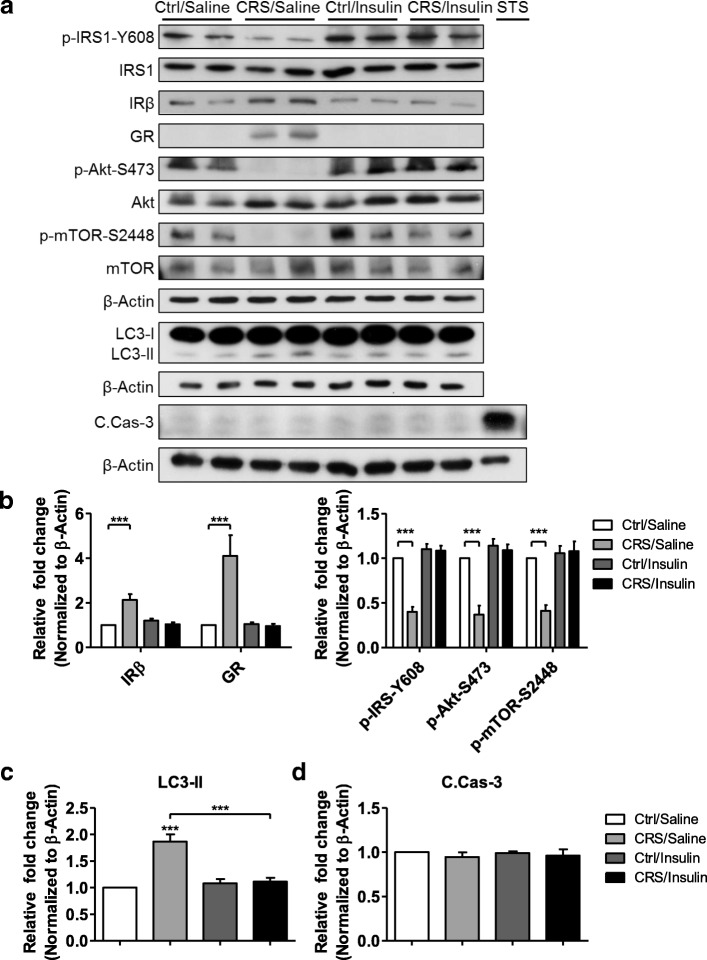
Prevention of CRS-induced hippocampal insulin signaling impairment and autophagy by intranasal insulin delivery. **a** Western blotting analysis of the insulin signaling pathway and the characteristics of cell death in the mouse hippocampus. **b** Quantification of each insulin signaling molecules analyzed by two-way ANOVA (IRβ, *n* = 9; GR, *n* = 7; p-IRS-Y608, *n* = 13; p-Akt-S473 *n* = 10; p-mTOR-S2448, *n* = 7). **c** Quantification of LC3-II analyzed by two-way ANOVA (*n* = 5 per group). **d** Quantification of C.Casp-3 analyzed by two-way ANOVA (*n* = 4 per group). Data are mean ± SEM. ***p < 0.001

We next measured the function of hippocampal spatial working memory by using the Y-maze test. Rodents typically prefer to explore a new arm of a maze instead of going to the previously visited arm. The Y-maze test measures the willingness of mice to explore new environments by counting the number of arm entries and calculating the percentage of alternation, which is related to the hippocampal spatial working memory. Normal mice show a high percentage of alternation. Thus, this test can easily quantify the cognitive deficits in mice [62, 63]. The number of total arm entries was similar in all four groups of mice, showing intact general locomotor activity (Fig. 5f, top). However, the CRS/Saline group showed a significantly reduced percentage of alternation compared to the Ctrl/Saline group, while the CRS/Insulin group recovered it to a level similar to those of Ctrl groups (Fig. 5f, bottom). To confirm biochemically whether intranasal insulin delivery recovered insulin signaling impaired by CRS, we measured the expression and phosphorylation levels of the relevant signaling proteins in the hippocampus by immunoblotting analysis (Fig. 6a). The expression levels of GR and IRβ were increased only in the CRS/Saline group (Fig. 6b, left). Interestingly, the levels of p-IRS1-Y608, p-Akt-S473, and p-mTOR-S2448 were reduced in the CRS/Saline groups but recovered in the CRS/Insulin groups (Fig. 6b, right). Altogether, these data suggest that intranasal insulin delivery relieved stress in the hippocampus.

Previous study reveals that insulin withdrawal causes a caspase-independent autophagic cell death in adult hippocampal neural stem cells [48], and we confirmed CORT impairs insulin signaling and induces autophagy features in mouse hippocampal neurons in Figs. 1 and 2. We next examined whether CRS-induced impairment of insulin signaling induced the feature of apoptosis or autophagy. LC3-II levels in the hippocampal lysates were increased by CRS, which were suppressed by intranasal insulin administration (Fig. 6c). However, the level of C.Casp-3 was not significantly different between Ctrl and CRS mice hippocampus regardless of intranasal administration of insulin (Fig. 6d). STS-treated in vitro hippocampal neuronal lysate was loaded as a positive marker of C.Casp-3 (Fig. 6a, last lane).

In summary, our data demonstrate that, although psychological stress impairs hippocampal functions and insulin signaling, intranasal insulin delivery can efficiently prevent CRS-induced insulin signaling impairment and rescue deficits in hippocampus-dependent neurobehavior.

## Discussion

Here, we demonstrated that CORT induces cell death in mouse hippocampal neurons with the characteristics of autophagy rather than apoptosis. CORT was neurotoxic and impaired insulin signaling; namely, it reduced the levels of p-IRS1-Y608, p-Akt-S473, and p-mTOR-S2448 and increased the expression of IRβ. We also observed that CRS impaired insulin signaling in the mouse hippocampus, as revealed by reduced levels of p-IRS1-Y608, p-Akt-S473, and p-mTOR-S2448. Of interest, intranasal insulin delivery to CRS mice restored impaired insulin signaling and rescued hippocampal cognitive deficits, indicating that dysregulated insulin signaling underlies defective hippocampal function following psychological stress.

It remains elusive how CORT causes neuronal damage. CORT reportedly induces apoptosis in primary hippocampal neurons [42, 64, 65], although the incidence of apoptosis in rodent chronic stress models is rare despite hippocampal volume shrinkage and neuronal loss after physiological stress [66–68]. Interestingly, we observed an increase in autophagy flux, but failed to detect activation of caspase-3 in CORT-treated neurons. Moreover, a pan-caspase inhibitor, Z-VAD-FMK, did not prevent cell death after CORT treatment (data not shown). Further studies using genetic knockout mouse models with neuron-specific deletion of autophagy genes will be required to prove the autophagic nature of CORT- and CRS-induced neurotoxicity.

The molecular mechanisms underlying the effects of CORT on insulin signaling in the hippocampus are poorly understood [38–40]. In this study, we revealed that CORT impaired insulin signaling in the hippocampus, which notably reduced the phosphorylation of IRS1 on Y608. This residue is considered as a PI3K-binding site [69]. Therefore, the Y608 residue of IRS1 may be a primary target of CORT in the insulin signaling cascade.

It is an interesting question why insulin signaling is impaired in the hippocampus under psychological stress. Acute psychological stress using inescapable foot shock has no effect on the IR–IRS1–Akt pathway in the brain [70]. Therefore, different models of stress as well as duration and strength of stress could yield different outcomes in terms of hippocampal insulin signaling.

Moosavi et al. demonstrated that CRS impaired spatial performance of mice in the Morris water maze and a high dose of insulin microinjected into the hippocampus prevented this deficit [41]. However, intrahippocampal insulin microinjection is invasive and may result in infection and secondary tissue damage. Here, we used a less aversive and non-invasive intranasal insulin delivery procedure and confirmed successful delivery of insulin to the hippocampus within a few hours and enhanced insulin signaling in the hippocampus. Intranasal insulin administration also effectively recovered hippocampal functions, including spatial working memory and nesting behavior. This suggests that intranasal insulin administration can be a simple and convenient strategy for the therapy of neurodegenerative disorders such as Alzheimer’s disease which are also accompanied by the impairment of insulin signaling. Furthermore, intranasal insulin delivery in CRS-treated mice reduced the loss of body weight, suggesting additional benefits for other hippocampal functions, although this may be an indirect effect. On the basis of these results, we suggest that intranasal insulin delivery to the hippocampus can be applied to various neuropsychiatric disorders and neurodegenerative conditions, as it is effective for stress relief and neuroprotection, and is less aversive and easy to apply, although there is a possibility that insulin may act in other brain regions as well as hippocampus.

Although we have demonstrated that CORT impairs insulin signaling in hippocampal neurons in vitro, we have not confirmed whether CRS impairs insulin signaling in hippocampal neurons in vivo, as hippocampal tissue contains many types of cells including neural stem cells, neurons, and glia. Hence, it needs to be evaluated in which types of hippocampal cells insulin signaling is affected by CRS. Other unanswered questions include whether autophagy occurs in hippocampal neurons in vivo or how IRS1 phosphorylation on -Y608 is regulated. Nevertheless, our results indicate the potential involvement of autophagy in psychological stress-induced neuropathology and the effectiveness of insulin for neuroprotection and cure of hippocampal cognitive dysfunction.

## Abbreviations

ACTH: Adrenocorticotropic hormone
Baf.A1: Bafilomycin A1
BSA: Bovine serum albumin
C.Casp-3: Cleaved caspase-3
CORT: Corticosterone
CRH: Corticotropin-releasing hormone
CRS: Chronic restraint stress
Ctrl: Control
DTT: Dithiothreitol
GR: Glucocorticoid receptor
IR: Insulin receptor
IRS: Insulin receptor substrate
IRβ: Insulin receptor subunit β
LC3: Microtubule-associated protein light chain 3
mTOR: Mammalian target of rapamycin
PBS: Phosphate buffered saline
PFA: Paraformaldehyde
PI: Propidium iodide
PI3K: Phosphatidylinositol-3-kinases
PMSF: Phenylmethanesulfonyl fluoride
RIPA buffer: Radioimmunoprecipitation assay buffer
SEM: Standard error of mean
STS: Staurosporine
